# Tuning and Enhancing Quantum Coherence Time Scales in Molecules via Light-Matter Hybridization

**DOI:** 10.1021/acs.jpclett.2c02877

**Published:** 2022-12-05

**Authors:** Wenxiang Hu, Ignacio Gustin, Todd D. Krauss, Ignacio Franco

**Affiliations:** † Materials Science Program, University of Rochester, Rochester, New York14627, United States; ‡ Department of Chemistry, University of Rochester, Rochester, New York14627, United States; ¶ Institute of Optics, University of Rochester, Rochester, New York14627, United States; § Department of Physics, University of Rochester, Rochester, New York14627, United States

## Abstract

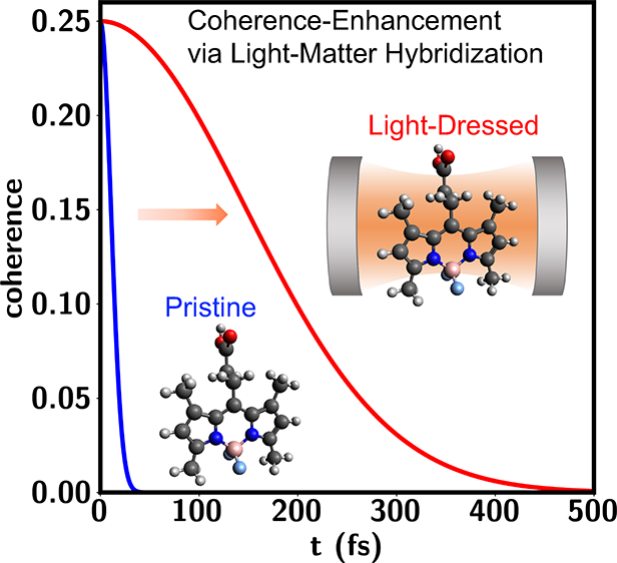

Protecting quantum coherences in matter from the detrimental effects introduced by its environment is essential to employ molecules and materials in quantum technologies and develop enhanced spectroscopies. Here, we show how dressing molecular chromophores with quantum light in the context of optical cavities can be used to generate quantum superposition states with *tunable* coherence time scales that are longer than those of the bare molecule, even at room temperature and for molecules immersed in solvent. For this, we develop a theory of decoherence rates for molecular polaritonic states and demonstrate that quantum superpositions that involve such hybrid light-matter states can survive for times that are orders of magnitude longer than those of the bare molecule while remaining optically controllable. Further, by studying these tunable coherence enhancements in the presence of lossy cavities, we demonstrate that they can be enacted using present-day optical cavities. The analysis offers a viable strategy to engineer and increase quantum coherence lifetimes in molecules.

One of the greatest challenges for science and engineering in the 21st century is to harness the quantum features of matter to fuel the next technological revolution.^1−6^ Molecules, in particular, are highly compact and configurable quantum systems. They offer manifolds of energy levels in the UV/visible (electronic/vibronic), infrared (vibrational) and microwave (rotational) regions of the electromagnetic spectrum and, thus, the possibility of implementing quantum operations in a variety of time scales.

In spite of this promise, molecules are currently not primary candidates for quantum technologies. This is because the molecular quantum coherence—that enables desirable quantum features such as the ability to interfere, be controlled or entangle—is very sensitive to the unavoidable interactions of the molecule with its surrounding environment. Such interactions introduce decoherence processes that corrupt the desired time-evolution of the molecule and thus its controllability.^7−9^ In fact, electronic (∼10s fs) and vibrational (∼1000s fs) coherence loss in molecules is extraordinarily fast.^10−13^

To open the sophistication of chemistry in building complex molecular architectures to develop next generation quantum technologies, there is a critical need to identify methods to better isolate the molecule from its environment and preserve its quantum coherence.^14−23^ Protecting and manipulating molecular coherences is also key to unshackling the chemical process from the constraints of thermal Boltzmann statistics, as needed to enhance molecular function through coherence,^24^ to develop novel routes for the quantum control of chemical dynamics,^5,6^ and for the design of optical spectroscopies with enhanced resolution capabilities.^10,26,27^

Here we show how dressing molecular chromophores with quantum light in the context of optical cavities can be used to generate quantum superposition states with *tunable* coherence time scales that are superior to those of the bare molecule, even at room temperature and for molecules immersed in solvent (see Figure 1a). That is, that by hybridizing the molecular states with those of quantum light to create so-called molecular polaritons^28−36^ it is possible to effectively engineer and reduce the polariton-nuclear interactions that lead to coherence loss while still retaining the optical controllability of the pairs of states involved.

**Figure 1:**
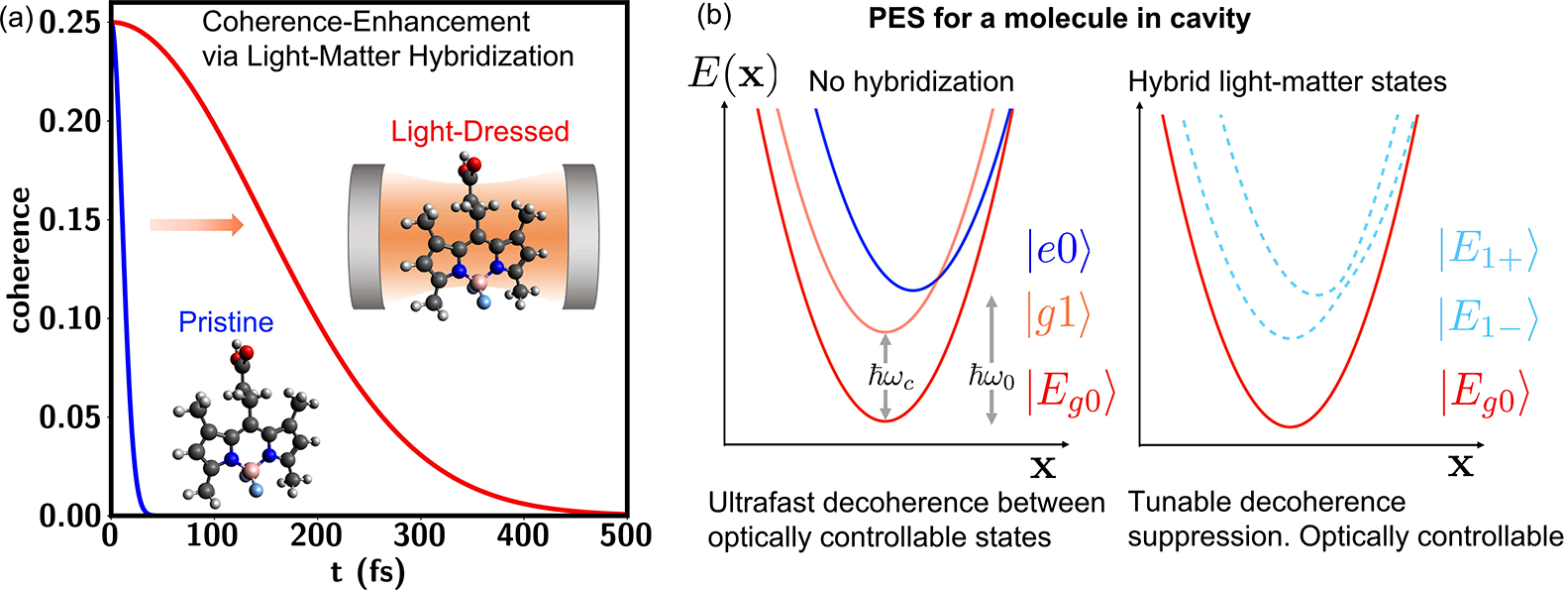
(a) Dressing molecular states with quantum light in optical cavities supports quantum superposition states with coherence time scales that are tunable and longer than those of the bare molecule. For example, (b) negative detunings of the cavity-photon resonance ℏω_*c*_, with respect to the molecular electronic excitation energy ℏω_0_, create a lower polariton state |*E*_1–_⟩ with a PES *E*(**x**) that is nearly parallel to the one of the ground-electronic/zero-photon state |*E*_*g*0_⟩ leading to long coherence times. Light-matter hybridization opens a nonzero transition dipole between these states via participation of the molecular excited/zero-photon state |*e*0⟩ that makes these superposition states optically controllable.

We focus on the coherence properties of a *single* molecular chromophore confined in a cavity, as experimentally realized by Baumberg et al.^37^ and, most recently, by Sandoghdar et al.^38^ Specifically, we develop a theory of decoherence time scales for polaritonic states in the condensed phase and demonstrate that such time scales can be enhanced by several orders of magnitude with respect to that of the bare molecule by dressing molecules with light, while retaining the optical controllability of the states. Further we characterize the influence of cavity losses on such coherence enhancements and demonstrate that the effect survives even for poor cavities.

We note that the effect identified here is distinct to the reduction of the reorganization energy that is observed when a molecular *ensemble* enters into strong coupling with an optical cavity mode. In that case, the polaritonic state is spread over many molecules and this leads to a reduction in the effective reorganization energy for the polaritons.^39−45^ For instance, Takahashi and Watanabe demonstrated^42^ that the dynamic inhomogeneity due to coupling of the lower polariton to a thermal environment inside the microcavity almost vanishes completely in this case. By contrast, the influence of light-matter hybridization on quantum coherence when a *single* molecule couples to a cavity remains largely unexplored. We demonstrate that this hybridization leads to coherence enhancements that become increasingly more important as the photonic component of the polaritons increases.

## Qualitative Physical Picture

For clarity, we first introduce the qualitative physical picture advanced by this study schematically summarized in Figure 1. As a measure of coherence in quantum dynamics, it is useful to follow the temporal decay of the off-diagonal elements of the system’s density matrix σ(*t*), σ_*nm*_(*t*) = ⟨ϕ_*n*_|σ(*t*)|ϕ_*m*_⟩ (*n* ≠ *m*), expressed in the eigenbasis {|ϕ_*n*_⟩} of the system’s Hamiltonian. In our case, the system will be the electronic and photonic degrees of freedom, and the decoherence will be introduced by interaction with the nuclei (solvent and vibrational modes).^7−9^ A well-known result of the theory of molecular electronic decoherence^14,15,46,47^ is that for early times *t* these coherences |σ_*nm*_(*t*)|^2^ = |σ_*nm*_(0)|^2^ exp(−*t*^2^/τ_*nm*_^2^) decay like a Gaussian with a time scale
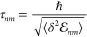
1dictated by the thermal and/or quantum fluctuations of the energy gap  with respect to its average value  at initial time. Here, *E*_*n*_(**x**) denotes the potential energy surface (PES) of state |ϕ_*n*_⟩ which depends parametrically on the nuclear (vibrational and solvent) coordinates **x**. That is,  =  where ρ_B_(0) is the density matrix of the nuclear environment at *t* = 0. While more general expressions for the decoherence time scales have been developed,^14,15^ this formula captures coherence decay for an initially pure system due to “pure-dephasing” processes where there are no transitions between the system eigenstates induced by the environment. Therefore, to enhance the coherence time, one seeks to reduce the fluctuations of the energy gap introduced by the nuclear environment. This requires finding or engineering pairs of states with parallel PESs, *E*_*n*_(**x**) = *E*_*m*_(**x**) + *k*_0_ where *k*_0_ is a constant offset, such that .

This is precisely what can be created by confining a molecule in an optical cavity. In this case, one can identify physical states |*E*_*g*0_⟩ ≡ |*g*0⟩ and |*g*1⟩ where the molecule is in the ground state *g* and there are 0 or 1 photons in the cavity (Figure 1b, left panel). These two states are energetically separated by the cavity photon energy ℏω_*c*_. Any interaction with solvent or other nuclear degrees of freedom {**x**} will change the energy of these states as determined by their PES. However, since the photon does not change the interactions between the molecular chromophore with its surrounding environment, these two PESs will be identical differing by a constant offset ℏω_*c*_. The physical consequence of this is that if a coherent superposition between these two states |Ψ⟩ = *c*_1_|*E*_*g*0_⟩ + *c*_2_|*g*1⟩ is created, its coherence will be robust to any quantum noise introduce by the thermal environment and limited solely by the cavity lifetime. This is in stark contrast with the coherences between that ground |*g*⟩ and excited |*e*⟩ state as the PESs of these two states are displaced in conformational space, and thus the thermal fluctuations of the environment lead to energy gap fluctuations and ultrafast coherence loss.

Unfortunately, these subspaces with protected coherences are inaccessible optically since the transition dipole between |*E*_*g*0_⟩ and |*g*1⟩ is zero due to the orthogonality of the photon states involved, making it impossible to control them using external laser sources.

To make these spaces optically controllable, we take advantage of the hybridization of light and matter. For definitiveness, consider the case in which the cavity frequency is negatively detuned from the optical transition of the molecule ℏω_0_, i.e. δ_*c*_ = ℏω_*c*_ – ℏω_0_ < 0, as shown schematically in Figure 1b. In the cavity |*g*1⟩ hybridizes with state |*e*0⟩ describing the molecule in excited state *e* and 0 photons in the cavity to produce an upper |*E*_1+_⟩ and lower |*E*_1–_⟩ polariton (dashed lines). Because the polaritonic states now have contributions from both |*g*1⟩ and |*e*0⟩, they now support a nonzero transition dipole with the ground state |*E*_*g*0_⟩ making this space optically controllable. As the cavity detuning δ_*c*_ is made more negative, the PES of |*E*_1–_⟩ becomes increasingly closer to that of |*g*1⟩, increasing the coherence time between |*E*_1–_⟩ and |*E*_*g*0_⟩. These coherences are always longer than that of the bare molecule as the effective PES of |*E*_1–_⟩ has a reduced displacement in conformational space with respect to the ground state, leading to motional narrowing^31,37,48^ upon photoexcitation. However, the larger the negative detuning of the cavity, the weaker the transition dipoles between the two states and therefore their optical controllability. In practice, a balance needs to be struck between the coherence enhancement and optical controllability. These physical insights are formalized below.

## Theoretical Model

As a minimal but useful model, we employ the Holstein–Jaynes–Cummings model, which consists of a single molecule with two (ground and excited) multidimensional PESs coupled to a single-mode cavity.^33,49^ We use this model to investigate how the coupling between matter and quantum light impacts the onset of decoherence. For clarity, we first focus on a molecule with a single vibrational mode in a lossless cavity. We then extend the analysis to the case of many nuclear modes at finite temperature and lossy cavities.

The Hamiltonian of this tripartite (electrons-photons-nuclei) quantum system is of the form *H* = *H*_M_ + *H*_C_ + *H*_MC_, where *H*_M_ describes the molecule, *H*_C_ the cavity and *H*_MC_ their interaction. The molecular Hamiltonian
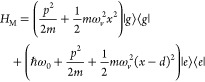
2consists of two electronic states {|*g*⟩, |*e*⟩} with a 0–0 transition ℏω_0_ and harmonic PESs that are displaced a distance *d* in conformational space. Here, *x*, *p*, *m*, and ω_*v*_ are the position, momenta, mass and frequency of the vibrational mode. The strength of the electron–nuclear coupling is determined by the dimensionless Huang–Rhys factor , which increases with the displacement *d*. The cavity consists of a single mode of frequency ω_*c*_ with Hamiltonian
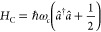
3where *â*^†^ and *â* are the photon creation/annihilation operators. The cavity-molecule interaction is taken in the rotating wave approximation where
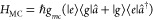
4This interaction assumes a “weak-resonance” condition where the cavity is near resonance to the electronic transition |ω_0_–ω_*c*_| ≪ ω_0_ and *g*_*mc*_ ≪ ω_0_.^49^

For a lossless cavity, the excitation can only be transferred from the molecule to the cavity or vice versa. Therefore, the dynamics with a fixed number of excitations is contained within the subspace {|*gn*_*c*_ + 1⟩, |*en*_*c*_⟩}, where *n*_*c*_ + 1 is the number of the photons in the cavity. We focus this analysis on the ground state |*E*_*g*0_⟩ and the first excitation subspace spanned by the {|*g*1⟩, |*e*0⟩} basis. For this, we project the Hamiltonian *H*_1_ = Π^†^*HΠ* using projection operator Π = |*E*_*g*0_⟩⟨*E*_*g*0_| + |*g*1⟩⟨*g*1| + |*e*0⟩⟨*e*0| to obtain
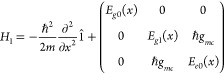
5where we have adopted the position representation for the nuclei and the {|*E*_*g*0_⟩, |*g*1⟩, |*e*0⟩} basis for the electron-photon component. Here, ,  =  and  are the (diabatic) PESs of the light-matter states in the basis. To obtain the lower and upper polaritonic PESs, we diagonalize the potential term in the equation above for each fixed nuclear geometry *x*. The resulting lower *E*_1,–_(*x*) and upper *E*_1,+_(*x*) polaritonic PESs are
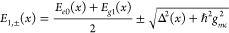
6where
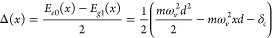
7and δ_*c*_ = ℏω_*c*_ – ℏω_0_ is the detuning of the cavity frequency with respect to the electronic energy gap. The associated lower |*E*_1–_⟩ and upper |*E*_1+_⟩ polaritonic states are given by
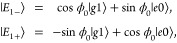
8where  and .

## Polaritonic Decoherence in the Condensed Phase

To obtain the decoherence times among light-matter states using eq 1, we need to evaluate . To do so, we focus on the weak electron–nuclear coupling limit and expand the transition energies  to first order in *d* around *d* = 0. This yields decoherence times of the form
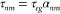
9where τ_*eg*_ = ℏ(*m*^2^ω^4^⟨δ^2^*x*⟩*d*^2^)^−1/2^ is the electronic decoherence time of the two-level molecule in the absence of the cavity.^15^ The quantity α_*nm*_ modulates the decoherence time of the pristine molecule and can be understood as a coherence enhancement factor due to the optical cavity. The coherence enhancements are given by
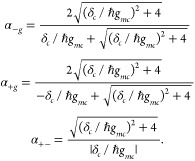
10Here α_–*g*_ and α_+*g*_ are the coherence enhancements between the ground state |*E*_*g*0_⟩ and the lower (−) and upper (+) polaritonic states |*E*_1,∓_⟩, and α_+–_ is that between the two polaritonic states. Note that the cavity can only enhance the quantum coherence time scale of the molecular system as α_*nm*_ ≥ 1.

The analysis can naturally be extended to the case in which the molecule is coupled to *N*_vib_ vibrational or solvent modes at finite temperature. For this, we take advantage that these *N*_vib_ nuclear degrees of freedom contribute independently to the decoherence at early times.^14,15^ That is, |σ_*nm*_(*t*)|^2^ = |σ_*nm*_(0)|^2^ exp(−∑_*k* = 1_^*N*_vib_^*t*^2^/(τ_*nm*_^(*k*)^)^2^) where τ_*nm*_^(*k*)^ is the decoherence time associated with the *k*th mode. As before, it is useful to describe these modes as harmonic oscillators of mass {*m*_*k*_} and frequency {ω_*k*_} that introduce displacements {*d*_*k*_} in the excited state PESs along coordinates **x** = {*x*_*k*_}. In this case, the coherence enhancements remain intact and the polaritonic decoherence time becomes
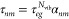
11where
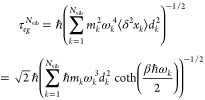
12is the decoherence time of the pristine molecule at temperature *T* ().^15^ At high temperatures (for which βℏω_*k*_ ≪1), the decoherence time scale becomes
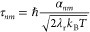
13where  is the reorganization energy (half the Stokes shift).

Equations 10–13 demonstrate that the quantum coherences of molecular systems in a cavity are enhanced with respect to those of the bare molecule. These enhancements survive even at room temperature and for molecules immersed in solvent and can be tuned by varying δ_*c*_/ℏ*g*_*mc*_. The latter can be accomplished by changing the cavity length and volume and by using molecules with different excitation frequencies and transition dipoles. The net influence of the cavity is to reduce the effective coupling of the (electrons + photon) system to its nuclear environment as measured by the reorganization energy λ_r_.

**Figure 2:**
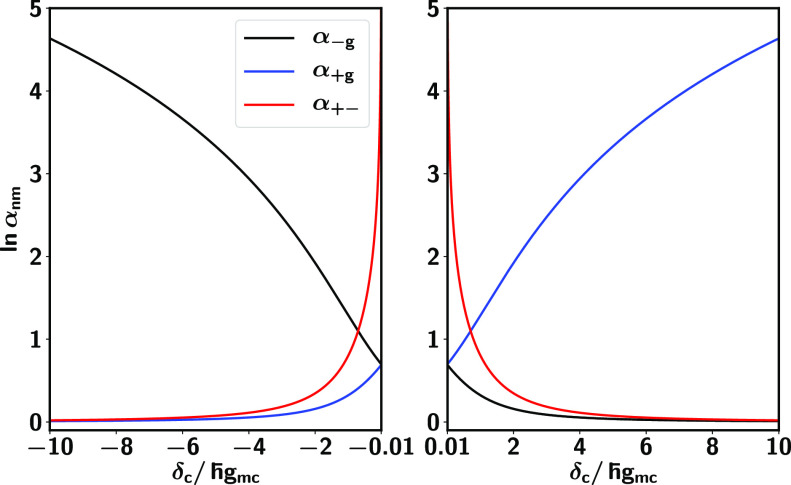
Cavity induced coherence enhancement as a function of δ_*c*_/ℏ*g*_*mc*_, where δ_*c*_ = ℏω_*c*_ – ℏω_0_ is the cavity detuning and ℏ*g*_*mc*_ the molecule-cavity coupling strength. The α_–*g*_ and α_+*g*_ are the coherence enhancements between the ground state |*E*_*g*0_⟩ and the lower (−) and upper (+) polaritonic states |*E*_1,∓_⟩, and α_+–_ is that between the two polaritonic states. Note that the dressing of molecular levels with quantum light can enhance the coherence time scales with respect to those of the bare molecule by several orders of magnitude for the three types of coherences investigated.

To understand the magnitude of the effect, consider Figure 2, which shows the dependence of the coherence enhancements α_*nm*_ on δ_*c*_/ℏ*g*_*mc*_, plotted on a logarithmic scale for all three cases. Overall, dressing the molecule with quantum light can dramatically enhance the lifetime of the molecular-based coherences by several orders of magnitude!

Each coherence is enhanced in a different parameter regime for the cavity detuning δ_*c*_ and cavity-molecule coupling strength ℏ*g*_*mc*_. Specifically, positive detunings for which δ_*c*_/ℏ*g*_*mc*_ > 0 increase the lifetime of coherences between the ground state |*E*_*g*0_⟩ and upper polaritonic state |*E*_1+_⟩. Negative detunings lead to enhanced coherences between the ground state and the lower polaritonic state |*E*_1–_⟩. In turn, the coherence enhancement between the two polaritonic states α_+–_ increases as |δ_*c*_/ℏ*g*| decreases and shows a singularity as δ_*c*_/ℏ*g* → 0 within the scope of applicability of the theory.

## The PES Perspective

It is instructive to rationalize the coherence enhancements by examining the shape of the PESs for the three states involved. For definitiveness, we illustrate the effect on a one-dimensional model with parameters meant to resemble BODIPY-2H, which is known to have a small reorganization energy and thus a relatively long electronic coherence time scale (τ_*eg*_ = 14.6 fs) at room temperature (*T* = 300 K). For this molecule, the energy gap is ℏω_0_ = 2.5 eV and the Stokes shift *s* = 0.064 eV.^50^ As a vibrational frequency we choose ω_*v*_ = 0.0902 fs^–1^, which corresponds to one of the vibrational modes being active during relaxation, and we assign an effective nuclear mass *m* = 459.3 eV fs^2^/Å^2^ based on (B3LYP 6-311++G(d,p)) density functional theory computations of the normal modes. The displacement *d* = 0.1307 Å is calculated from the Stokes shift *s* = 2λ_r_ = *mω*_*v*_^2^*d*^2^.

**Figure 3:**
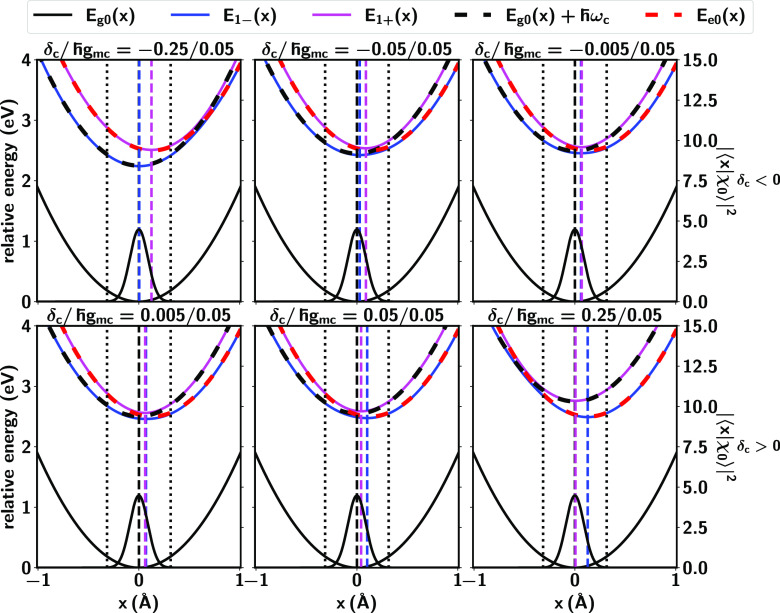
Change of PESs for the ground *E*_*g*0_(*x*), lower *E*_1–_(*x*) and upper *E*_1+_(*x*) polaritonic states for varying cavity detunings δ_*c*_ and fixed ℏ*g*_*mc*_ = 0.05 eV. The vertical dashed lines represent the nuclear equilibrium position of the PES with the same color. The initial nuclear wavepacket  of width *x*_*w*_ is plotted. The area between two vertical dotted lines covers the range [−2.5*x*_*w*_, 2.5*x*_*w*_].

Figure 3 shows the PES for varying δ_*c*_ but fixed ℏ*g*_*mc*_. By increasing δ_*c*_ the curvature and the nuclear equilibrium position for *E*_1+_(*x*) (magenta lines) become closer to that for *E*_*g*0_(*x*) (black lines), and thus the associated reorganization energy is reduced. This leads to the coherence enhancements between the ground state and the upper polariton seen in Figure 2 (blue line) with increasing δ_*c*_/ℏ*g*_*mc*_. The opposite change is observed for *E*_1–_(*x*) (blue lines in Figure 3). In this case, increasingly negative detuning makes the *E*_1–_(*x*) PES become increasingly parallel to *E*_*g*0_(*x*), leading to the increase in α_–*g*_ (black line, Figure 2). In turn, the coherences between the two polaritonic states are enhanced as |δ_*c*_| ≈ 0 because the curvature and the nuclear equilibrium position for two polaritonic surfaces become closer to each other as |δ_*c*_| → 0.

**Figure 4:**
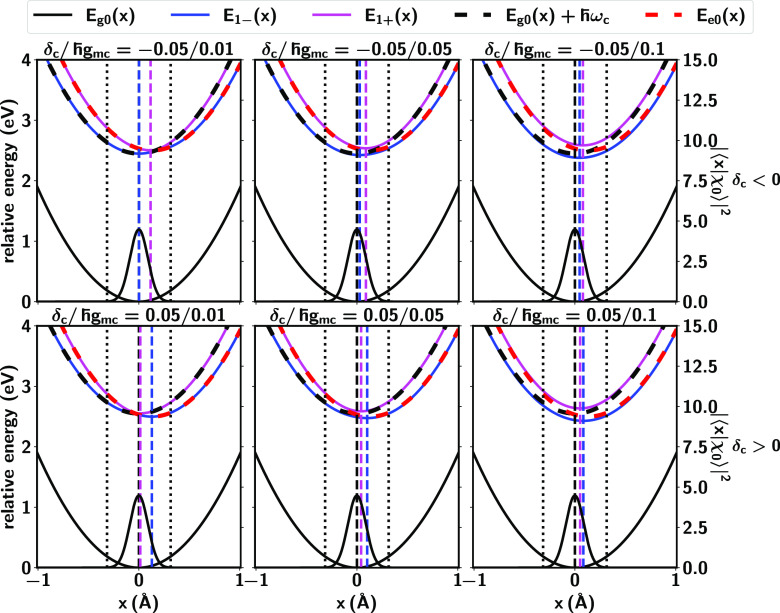
Change of PESs for varying molecule-cavity coupling strengths ℏ*g*_*mc*_ and fixed δ_*c*_ = −0.05 eV (top panels) and δ_*c*_ = 0.05 eV (bottom panels). The notation is identical to that in Figure 3.

Consider now Figure 4 (top panel), which shows the PESs for fixed negative detuning δ_*c*_ = −0.05 eV and varying ℏ*g*_*mc*_. As shown, weaker molecule-cavity coupling strength ℏ*g*_*mc*_ makes the nuclear equilibrium position for *E*_1–_(*x*) (blue lines) to be closer to that of *E*_*g*0_(*x*), and thus the enhancement α_–*g*_ increases. In turn, for fixed positive δ_*c*_ = 0.05 eV (Figure 4, bottom panel), decreasing ℏ*g*_*mc*_ makes the nuclear equilibrium position for *E*_1+_(*x*) (magenta lines) to be closer to that of *E*_*g*0_(*x*) and thus the α_+*g*_ enhancement increases.

These changes in the PES with varing δ_*c*_/ℏ*g*_*mc*_ can be understood by examining the molecular and photonic composition of the polaritons. The polaritons mix the |*g*1⟩ and |*e*0⟩ states [eq 8]. However, for larger negative (or positive) detunings the lower (or upper) polariton coincides with |*g*1⟩. This makes the shape of the PES for *E*_1–_(*x*) (or *E*_1+_(*x*)) increasingly similar to that of *E*_*g*0_(*x*) as δ_*c*_ ≪ 0 (or δ_*c*_ ≫ 0), thus reducing the entanglement with the nuclei that introduce decoherence. In turn, as |δ_*c*_| → 0,  ≈  and thus two polaritonic surfaces become approximately parallel to each other, thus suppressing the entanglement with the nuclear degrees of freedom.

## Optical Controllability

To take advantage of these states with enhanced coherences, it is necessary to be able to create and manipulate superpositions among them. This requires nonzero transition dipoles that enable excitation with coherent laser sources. From eq 8, the transition dipoles between {|*E*_*g*0_⟩, |*E*_1–_⟩ and |*E*_1+_⟩} are
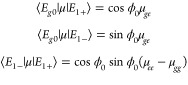
14where μ_*ij*_ = ⟨*i*|μ|*j*⟩ are matrix elements of the electronic dipole operator *i* = *g* and *e* and where we have adopted the Condon approximation. These transition dipoles are generally nonzero, making the space controllable through coherent laser sources. For spatially symmetric systems (as assumed in the Jaynes–Cummings model) μ_*ee*_ = μ_*gg*_ = 0 and there is no dipole transition between the lower and upper polaritons. Nevertheless, the ground and polaritonic states remain dipole connected even in this limit.

**Figure 5:**
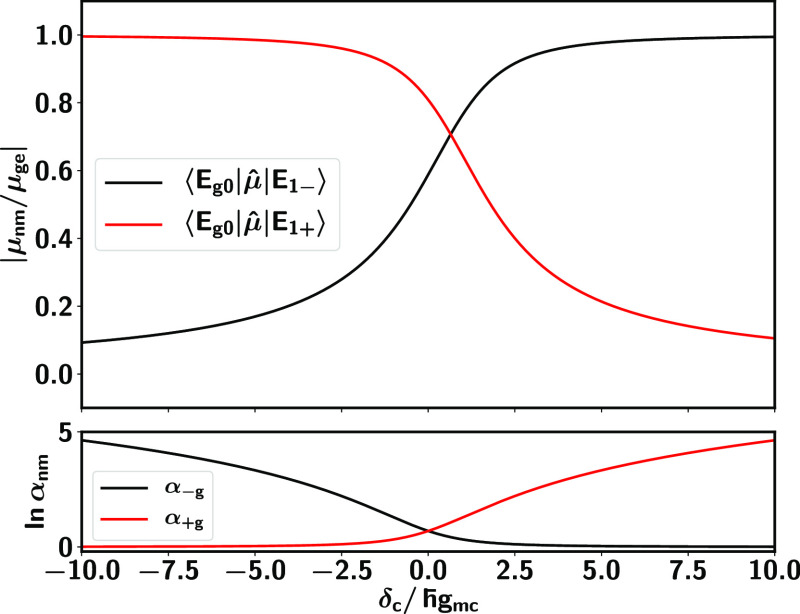
Transition dipoles (top panel) and corresponding coherence enhancement (bottom panel) in the hybrid system as a function of δ_*c*_/ℏ*g*_*mc*_ for ℏ*g*_*mc*_ = 0.05 eV. Nonzero transition dipoles between the polaritonic states and the ground state exist even when large coherence enhancements are achieved.

Figure 5 shows the dependence of the transition dipoles on δ_*c*_/ℏ*g*_*mc*_. We use the same parameters of Figure 3 where ℏ*g*_*mc*_ = 0.05 eV is fixed and the detuning δ_*c*_ varies from −0.5 to 0.5 eV. Further, we adopt the Condon approximation and take μ_*ee*_ = μ_*gg*_ = 0. As shown, the transition dipoles from |*E*_*g*0_⟩ to |*E*_1–_⟩ and |*E*_1+_⟩ are nonzero (top panel) in the range where large enhancement of coherence is achieved (bottom panel). Nevertheless, the process of enhancing the coherence time scales also reduces the magnitude of the transition dipoles between the states involved. The larger the enhancement, the weaker the transition dipole between the two states. This is because as the coherence enhancements become larger, the polaritonic state becomes increasingly closer to |*g*1⟩, which has a zero transition dipole with |*E*_*g*0_⟩. Thus, a balance must be struck between optical controllability and enhanced coherences.

The subspace with enhanced coherences will behave close to a two-level system (as opposed to a two-surface molecule) due to its reduced reorganization energy and is expected to exhibit Rabi oscillations upon resonant photoexcitation. Photoexcitation outside of this protected subspace can be minimized by choosing lasers with frequencies that are only at resonance with the desired transition.

## Lossy Cavities

Thus far the analysis is based on lossless cavities that do not allow photons to escape. However, in reality, the coupling between a cavity and the outside photonic environment causes the cavity photons to decay and lead to energy dissipation. From the quantum master equation for matter-cavity systems,^7,51,52^ this additional environment introduces an exponential decay of polaritonic coherences in time scales determined by the cavity lifetime τ_loss_.^7,53^ Thus, .

**Figure 6:**
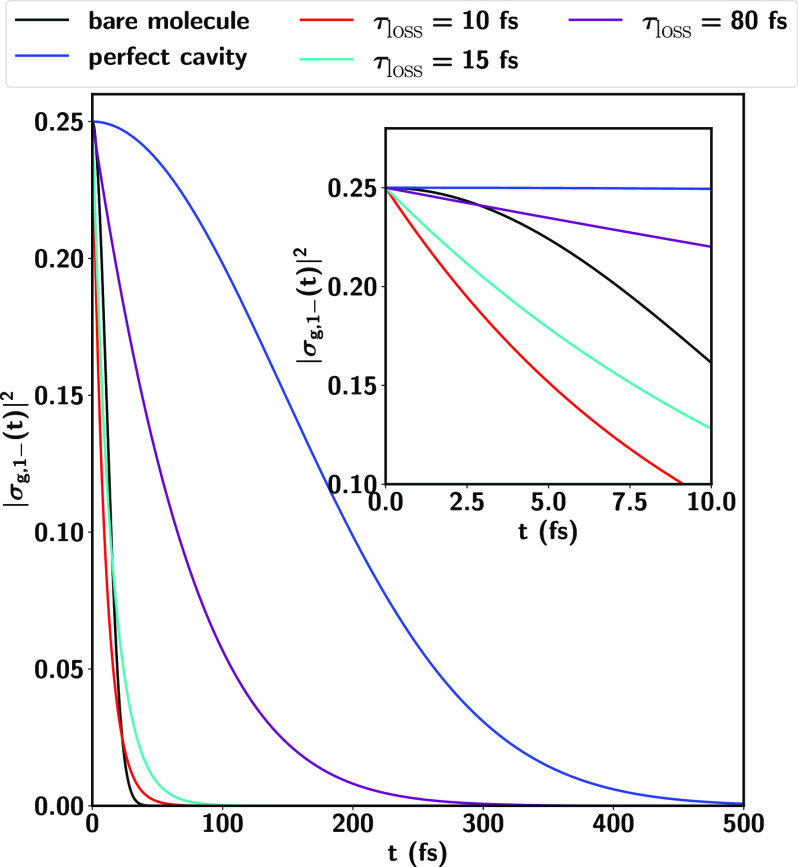
Coherence decay of an initial superposition  in a lossy cavity with varying lifetimes τ_loss_. The coherences in the bare molecule decay with a characteristic time scale of τ_*eg*_ = 15 fs. Note that the coherence enhancements partially survive even for cavities with lifetimes close to τ_*eg*_. The initial nuclear wave packet is defined in Figure 3.

To understand the quantitative influence of lossy cavities on the coherence enhancements, consider Figure 6, which contrasts the coherence decays for different cavity lifetimes τ_loss_ = 10, 15, and 80 fs with that of a perfect cavity (1/τ_loss_ = 0) for an initial superposition of the form  = . The computations assume δ_*c*_ = −0.15 eV and ℏ*g*_*mc*_ = 0.05 eV, for which the coherence enhancement is α_–*g*_ = 11.9. As shown, while for the bare molecule the coherences decay in ∼15 fs, an order of magnitude enhancement in the coherence lifetime is observed for the same molecule in a cavity. Cavity losses reduce, but do not eliminate, the effective coherence enhancements. Even for a relatively poor cavity with τ_loss_ = 80 fs, the coherent superpositions of light-matter states survive for hundreds of fs. When τ_loss_ is comparable with the decoherence time for the bare molecule (τ_loss_ = 10 and 15 fs), at early times the coherence decay is faster, as both molecular and cavity environments contribute to the loss but, surprisingly, the overall coherence decay rate in the hybrid system is still slower than that of the bare molecular system. For cavities with lifetimes τ_loss_ ≫τ_*nm*_ full coherence enhancements are observed.

For a given cavity frequency ω_*c*_ the cavity lifetime increases with the quality *Q* = ω_*c*_τ_loss_ factor.^54^ Experimentally, high *Q* factors can be achieved for Fabry–Perot (*Q* ∼ 2000) and photonic crystal (*Q* ∼ 13000)^55^ cavities. Even for lossy plasmonic cavities *Q* can be as high as 1376.^56^ These high *Q* factors provide cavity photon lifetimes from few hundreds of femtoseconds to picoseconds (assuming ℏω_*c*_ ∼ 2.5 eV as used in the examples above). By contrast, the typical electronic coherence lifetime in molecules is approximately tens of fs. This implies that present day cavities can lead to a full enhancement of molecular coherences due to dressing with quantum light.

Further note that while the Purcell effect can enhance the rate of spontaneous emission by a factor ,^57^ the effective rates of spontanenous emission are still expected to be much slower than the decoherence rates even in the limit of small cavities with a volume *V* ∼ λ of the same order of magnitude of the wavelength of light λ for which *F* ∼ 100 for *Q* ∼ 1500.

## Conclusion

The possibility of enhancing and engineering quantum coherences by dressing matter with quantum light offers exciting opportunities to unlock chemistry for quantum technologies. In this paper we have demonstrated that the hybridization of molecules with quantum light is a viable strategy to create optically controllable quantum subspaces with tunable and enhanced coherence time scales. For this we developed a theory of decoherence time scales for polaritonic states and demonstrated that the effect of the light-matter hybridization is to reduce the effective reorganization energy for single molecules while partially retaining the optical controllability of the states. Such coherence enhancements can increase the coherence time scales of molecules by several orders of magnitude. However, the larger the coherence enhancements, the smaller the transition dipole between the states involved, and thus a balance must be struck between optical controllability and enhanced coherence.

The analysis is based on a regime of the light-matter interaction in which a single molecule is near strong coupling with a cavity as realized in plasmonic^37^ and Fabry–Perot cavities.^38^ Further, the analysis assumes small reorganization energies and focuses on the pure dephasing component of the decoherence. As detailed in the Supporting Information, this pure dephasing limit can be satisfied in the regime where large coherence enhancements are expected. When the pure dephasing approximation is not applicable, the physical idea behind the coherence enhancements becomes of narrower applicability as the polariton-nuclear interactions will generate transitions into states for which no such enhancements exist. Further, the identified phenomenon requires quantum light with a fixed number of photons (Fock states), as opposed to classical coherent states of light with a Poisson distribution of the occupation of the Fock states.

The isolated phenomenon is expected to be general and applicable to electronic, vibronic, torsional and vibrational degrees of freedom. Future prospects include identifying possible coherence enhancements that can be enacted by dressing with classical light,^58^ studying the effect from the perspective of a full cumulant expansion,^59^ and capturing the coherence enhancements for molecules with strong electron–nuclear couplings.

## Supporting Information Available

Computational simulations testing the validity of the pure dephasing limit in molecular polaritons and figures showing population dynamics for various initial states (PDF)
