# Supplementary material for: Tuning and Enhancing Quantum Coherence Time Scales in Molecules via Light-Matter Hybridization
Source: J Phys Chem Lett. 2022 Dec 5;13(49):11503–11. doi: 10.1021/acs.jpclett.2c02877 (PMC9761670; doi:10.1021/acs.jpclett.2c02877)
Supplement: Supplementary file 1 — jz2c02877_si_001.pdf [file jz2c02877_si_001.pdf]

Supplementary Information: Tuning and Enhancing Quantum Coherence Time Scales in Molecules via Light-Matter Hybridization

Wenxiang Hu,[1] Ignacio Gustin,[2] Todd D. Krauss,[2,3] and Ignacio Franco[2,4]

[1] *Materials Science Program, University of Rochester, Rochester, New York 14627, USA*
[2] *Department of Chemistry, University of Rochester, Rochester, New York 14627, USA*
[3] *Institute of Optics, University of Rochester, Rochester, New York 14627, USA*
[4] *Department of Physics, University of Rochester, Rochester, New York 14627, USA* *

(Dated: November 17, 2022)

VALIDITY OF THE PURE DEPHASING LIMIT

The theory of coherence enhancements due to light-matter hybridization is based on the pure dephasing limit where there is no nuclear-induced population transitions between the polaritonic states. To test this approximation we follow the quantum dynamics of the polaritonic states under varying conditions for the one-dimensional model for BODIPY-2H used to exemplify the theory in Fig. 3 and 4 in the main text. We show that the pure dephasing limit can be accurate in regimes where strong coherence enhancements are expected.

Quantum dynamics simulations below are performed using the multi-state split operator method using a propagation time step $\Delta t = 0.001$ fs and a spatial grid defined in the $[-2, 2]$ Å range with $N = 2048$ grid points. Computations were tested for convergence on the time step, grid range and spacing.

The polaritonic adiabatic states are obtained by diagonalizing the Hamiltonian

$$H_1' = -\frac{\hbar^2}{2m}\frac{\partial^2}{\partial x^2}\hat{\mathbf{1}} + \begin{pmatrix} E_{g1}(x) & \hbar g_{mc} \\ \hbar g_{mc} & E_{e0}(x) \end{pmatrix}, \tag{1}$$

expressed in the $\{|g1\rangle, |e0\rangle\}$ diabatic basis for each fixed nuclear geometry x. This procedure yield eigenstates

$$\begin{aligned} |E_{1-}(x)\rangle &= \cos\phi(x)|g1\rangle + \sin\phi(x)|e0\rangle, \\ |E_{1+}(x)\rangle &= -\sin\phi(x)|g1\rangle + \cos\phi(x)|e0\rangle, \end{aligned} \tag{2}$$

where the mixing angle $\phi(x)$ is

$$\begin{aligned} \sin(2\phi(x)) &= -\hbar g_{mc}/\sqrt{\Delta^2(x) + \hbar^2 g_{mc}^2}, \\ \cos(2\phi(x)) &= \Delta(x)/\sqrt{\Delta^2(x) + \hbar^2 g_{mc}^2}. \end{aligned} \tag{3}$$

The polaritonic diabatic basis employed in the analysis is obtained by setting $x = 0$ in Eq. (2). In the pure dephasing limit, the population of the polaritonic diabatic states do not change and the diabatic and adiabatic picture should coincide. We use this criterion below to test the validity of the pure dephasing approximation.

Consider first a system prepared in state $|\Psi(0)\rangle = |E_{1-}\rangle|\chi_0\rangle$ where $|\chi_0\rangle$ is the ground vibrational state in the ground electronic state and $|E_{1-}\rangle$ is the diabatic lower polaritonic state. Figures S1 and S2 show the adiabatic (dashed lines) and diabatic (solid lines) populations for various $\delta_c/\hbar g_{mc}$. As shown, as $|\delta_c|$ or $\hbar g_{mc}$ increases the pure-dephasing approximation becomes increasingly accurate. This is because increasing $|\delta_c|$ or $\hbar g_{mc}$ increases the energetic separation between the lower and upper polaritonic states.

These results show that the main regimes of coherence enhancements that involve the lower polaritonic state are accessible in the pure dephasing limit. For example, for fixed $\hbar g_{mc} = 0.05$ and $\delta_c \leq -0.15$ ($\delta_c/\hbar g_{mc} \leq -3$), approximately stationary diabatic/adiabatic population dynamics is observed. That indicates that the pure dephasing limit is valid in the region $\delta_c/\hbar g_{mc} \leq -3$ where coherence enhancement $\alpha_{-g} \geq 7$ is achieved. Even the regime of $\delta_c/\hbar g_{mc} \to 0$ that exhibits large α_{+-} coherence enhancements can be accessed in the pure dephasing limit (see Fig. S2 right panels) by increasing $\hbar g_{mc}$.

Figures S3 and S4 detail the population dynamics for an initial state $|\Psi(0)\rangle = |E_{1+}\rangle|\chi_0\rangle$. In this case also, this dynamics becomes more pure dephasing as δ_c and $\hbar g_{mc}$ increase. The pure dephasing limit is more challenging to obtain because the larger initial force on the initial nuclear wavepacket in $E_{1+}(x)$ leads the nuclear wavepacket to move toward the avoided crossing area between the two polaritonic surfaces. Nevertheless, it is still possible to access the regimes of large coherence enhancements in the pure dephasing limit. For example, for $|\delta_c/\hbar g_{mc}| \sim 5$ with $\hbar g_{mc} = 0.05$ eV for which a coherence enhancement $\alpha_{+g} \sim 20$ is obtained leads to a dynamics that is well described by a pure dephasing Hamiltonian.

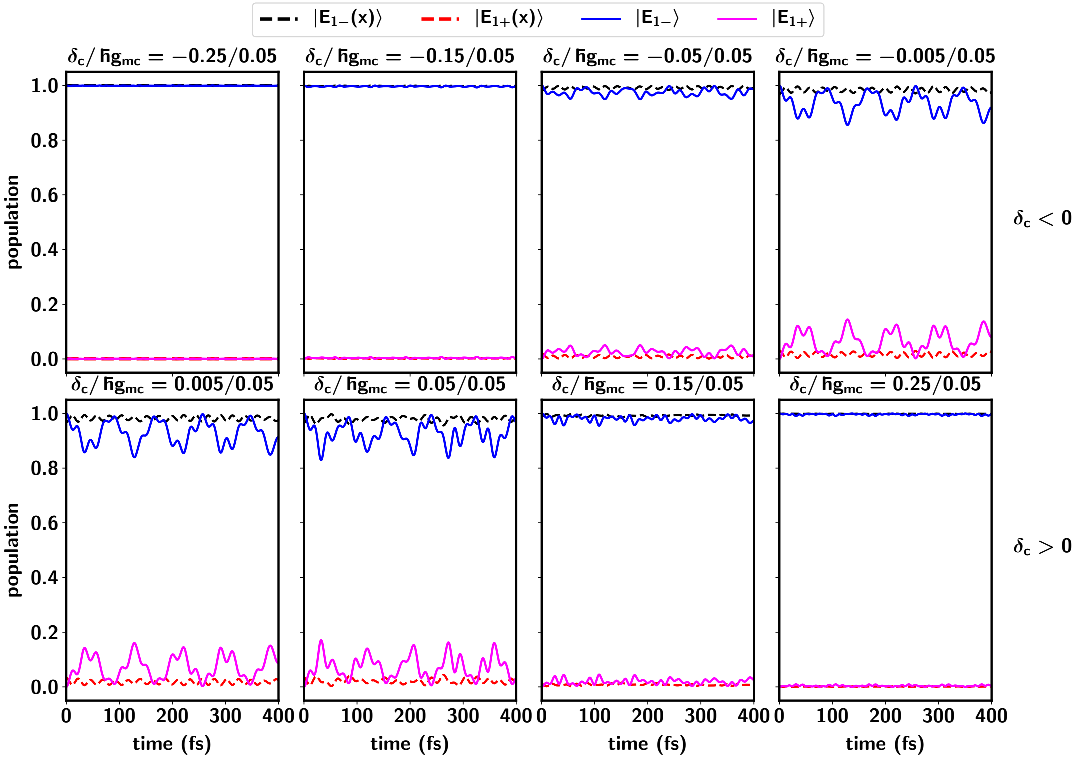

Figure S1. Population dynamics for an initial state $|\Psi(0)\rangle = |E_{1-}\rangle|\chi_0\rangle$ under various δ_c with fixed $\hbar g_{mc} = 0.05$ eV.

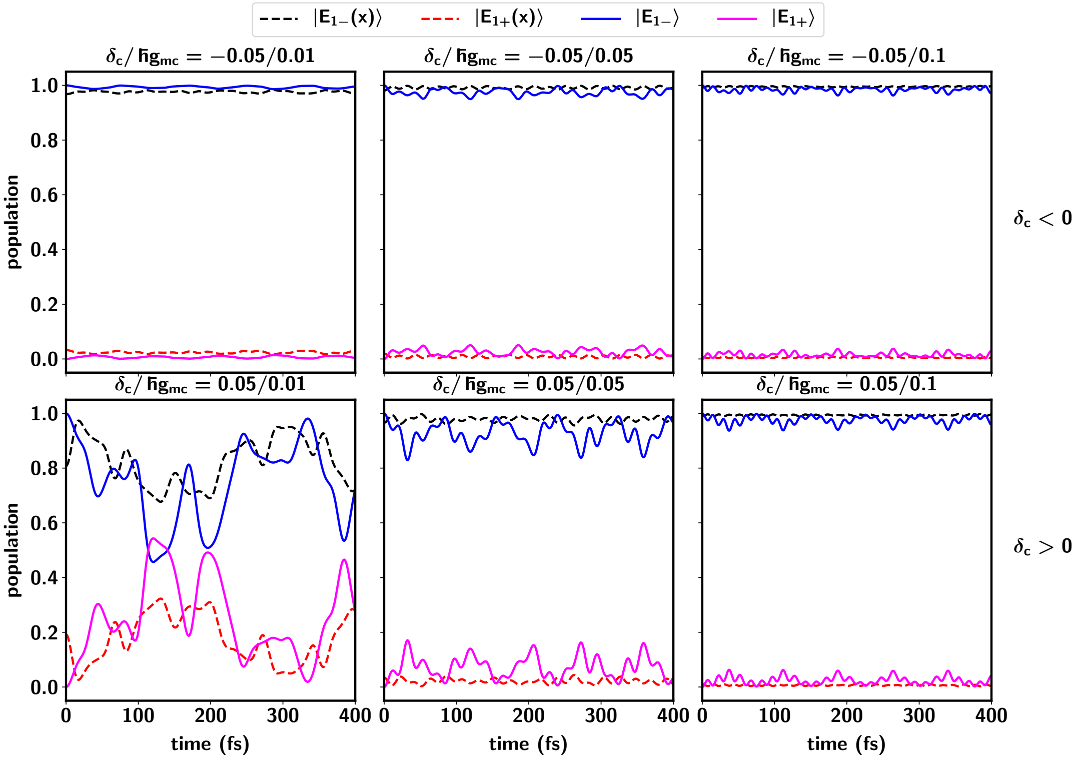

Figure S2. Population dynamics for an initial state $|\Psi(0)\rangle = |E_{1-}\rangle|\chi_0\rangle$ under various $\hbar g_{mc}$ with fixed $\delta_c = -0.05$ eV (first row) and $\delta_c = 0.05$ eV (second row).

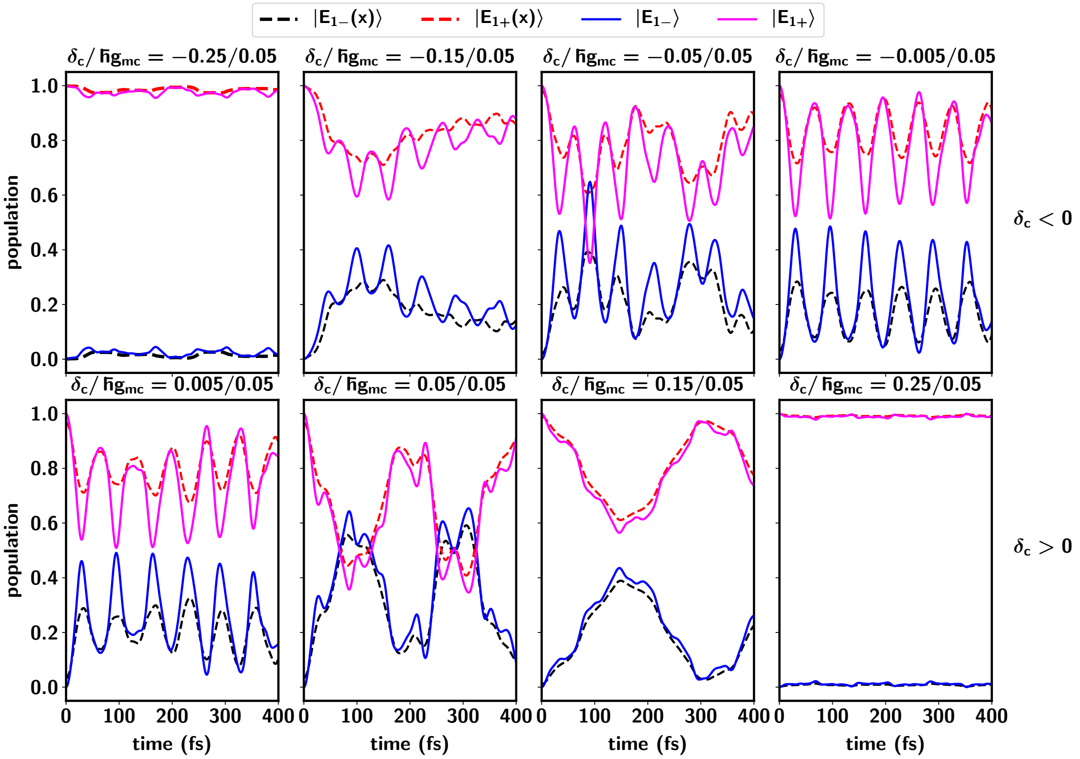

Figure S3. Population dynamics for an initial state $|\Psi(0)\rangle = |E_{1+}\rangle|\chi_0\rangle$ under various δ_c with fixed $\hbar g_{mc} = 0.05$ eV.

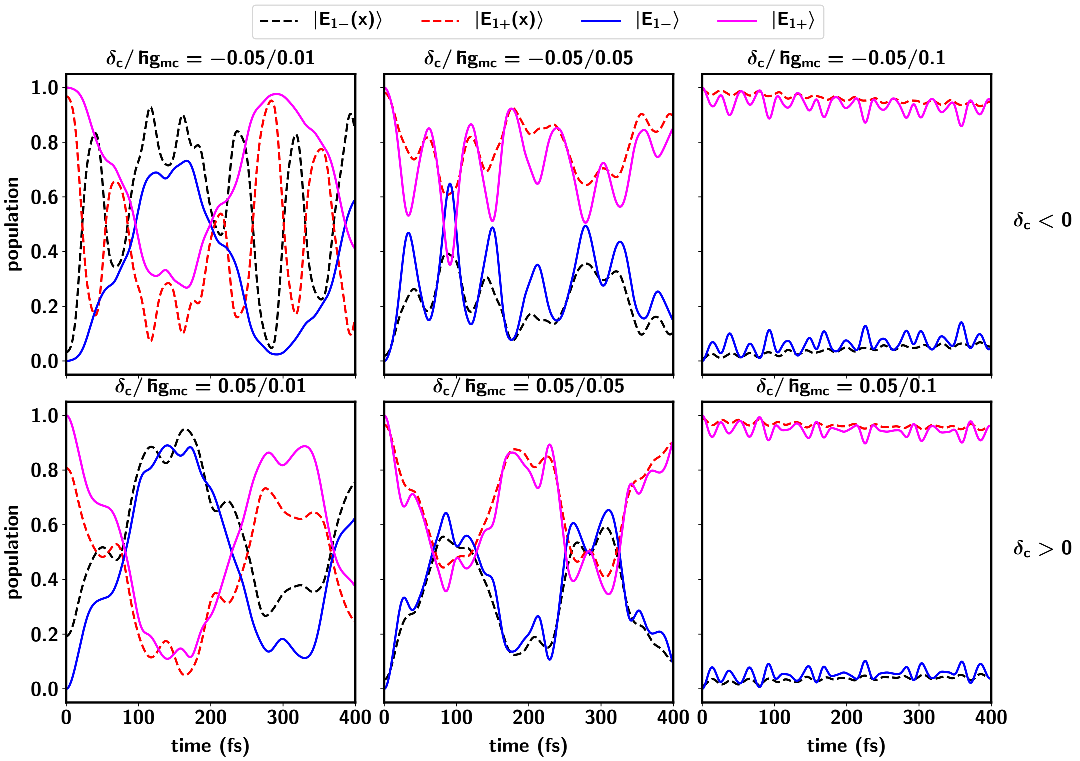

Figure S4. Population dynamics for an initial state $|\Psi(0)\rangle = |E_{1+}\rangle|\chi_0\rangle$ under various $\hbar g_{mc}$ with fixed $\delta_c = -0.05$ eV (first row) and $\delta_c = 0.05$ eV (second row).
